# Physical activity and its benefits on adolescents' mental health through self-esteem

**DOI:** 10.3389/frcha.2024.1503920

**Published:** 2024-12-16

**Authors:** Catherine Laurier, Katherine Pascuzzo, Vicky Jubinville, Annie Lemieux

**Affiliations:** ^1^ Department of Psychoeducation, Université de Sherbrooke, Sherbrooke, QC, Canada; ^2^ Groupe de Recherche et d’intervention sur les Adaptations Sociales de l’enfance (GRISE), Université de Sherbrooke, Sherbrooke, QC, Canada; ^3^ Institut Universitaire Jeunes en Difficultés (IUJD), Montréal, QC, Canada; ^4^ Centre de Recherche Charles-Le Moyne (CRCLM), Université de Sherbrooke, Longueuil, QC, Canada

**Keywords:** physical activity, mental health, self-esteem, adolescent, psychological distress, COVID-19

## Abstract

**Background:**

Since the onset of the COVID-19 pandemic, the scientific community has been concerned about the high rates of psychological distress among adolescents. The pandemic not only tested adolescents’ adaptation, but also disrupted key areas of their development. This demonstrates the need to study their psychological adjustment over time during this critical period to better guide services.

**Objective:**

This study sought to explore the extent to which physical activity and its association with self-esteem in the first months of the pandemic impacted adolescents' psychological distress, six months later.

**Methods:**

Two hundred and ninety-four adolescents (73% girls) between the ages of 12 and 17 participated in a longitudinal study launched at the start of the COVID-19 pandemic. The number of hours spent engaging in physical activity (HPA) in the past week and self-esteem were measured at Time 1 (T1; summer 2020). Psychological distress was measured at T1 and Time 2 (T2; winter 2021).

**Results:**

More HPA in the past week were related to greater self-esteem at T1. Greater self-esteem at T1 was related to lower psychological distress, six months later (T2). Lastly, HPA in the past week was not directly linked to psychological distress at T2, which confirms a fully indirect model.

**Conclusion:**

Results suggest that physical activity is a key factor in promoting better mental health adjustment through its benefits in terms of self-esteem, even during times of turmoil. Findings reinforce the recommendation of promoting the practice of sports and athletic activity in difficult times.

## 1 Introduction

The onset of the COVID-19 pandemic in 2020 launched global concern regarding the high rates of psychological distress among adolescents throughout the scientific community. While adolescent mental health was already of great interest long before the COVID-19 pandemic (1–3), the context of this global health crisis exacerbated its relevance. Indeed, due to the restrictive health measures implemented, social contacts were greatly reduced, testing adolescents' ability to adapt and challenging their psycho-emotional development (4–6). Considering this situation, researchers quickly recognized the necessity to better understand the potential short, mid, and long-term consequences of the COVID-19 pandemic on youth mental health to mitigate its negative impacts (7).

Exercise is known to reduce anxiety and stress symptoms (8, 9), psychological difficulties (10–13) as well as diminish symptoms of depression (14–16). Côté and Fraser-Thomas (17) have investigated the effects of physical activity on the development of young people in general. The most frequent benefit of physical activity is undoubtedly in the area of physical health (lifestyle habits, cardiovascular health, body weight, muscular strength and flexibility, etc.) (17, 18). Sports and physical activity also boost self-esteem and reduce stress, contributing to young people's emotional development and general well-being (17). A longitudinal study comparing inactive, beginner, and active individuals found that regular physical activity was positively associated with higher levels of happiness, self-esteem, and life satisfaction (19). Among adolescents, participation in sports and the development of athletic skills have been associated with lower levels of depression (20, 21) and decreased risks of depression and suicidal ideation, notably through improved self-esteem (22). Moreover, a study has shown that, among young adults, maintaining a daily routine and engaging in physical or sport activities were among the coping strategies associated with lower distress during the COVID-19 pandemic (23).

The links between physical activity interventions and mental health (24, 25) as well as psychosocial adjustment (externalizing problems, internalizing problems, self-concept, academic achievement, mental health disorders) (26, 27) are therefore well established, beyond the scope of the COVID-19 pandemic. To this end, the World Health Organization (WHO; 28) recommends that adolescents engage in moderate aerobic physical activity for an average of 60 min per day (7 hours a week). Yet, leisure-related physical activity among adolescents aged 12–17 years of age fell from an average of 27 min a day in 2018 to 20.3 min a day in 2020, equivalent to 3.15 h a week in 2018 and 2.4 h a week in 2020, well below the WHO recommendations (29). Indeed, research suggests that physical activity (activity time per week) among adolescents decreased, and sedentariness increased during the COVID-19 pandemic in relation to the health measures imposed (30). Although physical activity is recognized as beneficial for one's mental health, findings from the literature are nonetheless divided on dose and intensity required to obtain the associated benefits. Indeed, it is possible that when an individual reaches a certain level of physical activity in terms of performance requirements and time devoted to practice (e.g., recreational, competitive or elite sport), mental health benefits diminish as the pressure to perform increases (31, 32). The present study focuses on recreational participation in physical activities and sports.

A literature review conducted in the early months of the COVID-19 pandemic (12) and including 21 studies found that people who engaged in regular physical activity, in terms of duration and frequency, had fewer symptoms of depression and anxiety. This is also the conclusion reached by a review of the literature on the links between physical activity and mental health during the COVID-19 pandemic, which included 23 studies conducted in China in 2020 and 2021 (33). For adolescents specifically (13–18 years), a literature review (34) examined changes in physical activity after the onset of the COVID-19 pandemic and their impact on well-being. Combining 15 studies, the authors concluded that greater physical activity was associated with greater well-being, altered eating habits and free time, and an increase in obesity and internalizing symptoms (including anxiety and depression). Another recent literature review (35), based on 14 studies, reported on the links between physical activity and psychological or behavioral problems in children and adolescents during the COVID-19. Overall, a significant protective effect of physical activity was found on depression, anxiety, stress, mood problems, and behavioral problems such as irritability, problems with friends, conduct problems, hyperactivity or inattention, and prosocial behaviors.

In the same vein, an initial study by our research team found that adolescents who continued to engage in physical activity in the early months of the COVID-19 pandemic were less likely to experience psychological distress compared to those who stopped their activities (36). This is consistent with the results of a study conducted in Brazil during the early months of the COVID-19 pandemic. The authors reported that physical activity (more than 30 min of moderate activity or more than 15 min of vigorous activity per day) was associated with less symptoms of depression and anxiety, whereas a sedentary lifestyle (sitting more than 10 h a day) was associated with more symptoms of depression and anxiety (37). A Chinese study (38) also found a link between higher levels of physical activity in the past 7 days and more positive mood scores (less tension, depression, anger, fatigue, and confusion and more vigor and self-esteem) in adolescents during the COVID-19 pandemic.

While these studies add significantly to our understanding of the benefits of physical activity during the COVID-19 pandemic, their findings are mostly cross-sectional such that the effects of physical activity on adolescents' mental health over time during the pandemic are scarce. Assessing the effects of physical activity on young people's mental health six months following the onset of the COVID-19 pandemic is particularly pertinent as the uncertainty related to this unprecedent crisis and associated restrictions persisted and even spiked in certain regions, namely Quebec. As such, it is imperative that further research shed light on the factors that protect against worsening distress over time.

To this end, a Canadian study (39) with four measurement times from April 2020 (T1) to summer 2021 (T4) found that higher levels of vigorous activity across the COVID-19 pandemic (averaged across the four time points) was a significant negative predictor of depression, as well as anxiety, at T4. Higher activity levels also promoted greater self-esteem at T4. Though Covid-19 related stress at T1 predicted greater feelings of loneliness, depression, anxiety and lower self-esteem at T4, adolescents' engagement in physical activity buffered against anxiety and was related to greater self-esteem at T4. In addition to shedding light on the longitudinal association between physical activity and psychological adjustment during the COVID-19 pandemic, the findings also underscore the benefits of physical activity on self-esteem.

In general, self-esteem is recognized as a protective factor against psychological distress (40) and can be promoted by sport participation (22, 31). A study from the United Kingdom reported on the protective effect of physical activity on perceived stress through self-esteem as a mediating variable (38). The practice of physical activity was found to be positively related to better self-esteem, which in turn was associated with less perceived stress and less anxiety and depression (41). An additional study (39) also put forward this association between sports practice and lower depression in 14- and 15-year-old adolescents through greater self-esteem. These ﬁndings support that physical activity may be indirectly related to perceived stress, and anxiety/depressive symptoms through self-esteem.

Overall, previous research supports that physical activity is related to greater mental health and self-esteem and that these associations held true during the COVID-19 pandemic. Yet, findings rely largely on concurrent data, and none assess the effects of greater physical activity at the start of the pandemic on later psychological distress, controlling for initial levels of distress. Moreover, while some research suggests a potential mechanism through self-esteem (22, 41, 42), none of these have investigated these associations using a longitudinal design. Does increased physical activity contribute to a reduction in psychological distress throughout the COVID-19 pandemic? Is self-esteem a potential underlying mechanism in this relationship? The following study aims to shed light on these important questions.

### 1.1 Objectives

The first aim of this paper is to explore the extent to which physical activity and self-esteem in the first months of the COVID-19 pandemic are related to adolescents' psychological distress six months later, while controlling for initial levels of distress at the start of the pandemic. The second aim is to explore the possible indirect role of self-esteem in the expected association between greater physical activity and lower psychological distress, while controlling for initial distress.

## 2 Method

### 2.1 Recruitment

Adolescents were recruited between June and August 2020 via social media (Facebook, Instagram) posts and invited to participate in an online study seeking to better understand youth experiences during the COVID-19 pandemic. The link to access the questionnaire was available on the study's Facebook page, published on the researchers' university website. Facebook advertisements were purchased to increase the study's visibility among adolescents between the ages of 14 and 17 from Quebec. Facebook is a cost-effective recruitment strategy to promote sample representativity (43). For those under the age of 14, parents first consented to their adolescent's participation and then provided their adolescent's email address via an online questionnaire.

Adolescents’ participation in the study was voluntary and consent was obtained before access to the online survey. Participants took approximately 45 min to complete the survey, and those who did were entered into a draw for a chance to win one of ten 50$ gift cards. The human participant procedures were reviewed and approved by the ethics committees of the University of Sherbrooke.

The first measurement time took place in the summer of 2020 (June - August 2020, Time 1; T1) and the second measurement time took place in the winter of 2021 (January - March, Time 2; T2). At T1, several restrictive health measures were in place, including the closure of sport and activity centers. Several schools were also closed from spring 2020 until the end of the school year, with contact between peers restricted. In winter 2021 (T2), while most students returned to school, older high school students alternated between in-person and online classes to limit the spread of the virus. Several restrictions, such as mask wearing in public places, were also still enforced, further restricting the possibility of engaging in sport activities. As such, T1 is not a pre-pandemic measure. It took place after the first few months of the pandemic, when restrictive measures were very present, impacting social relations and restricting normal activities. T2 took place when the population had resumed many of its usual activities, but with numerous restrictive measures still in place.

### 2.2 Participants

This study included 294 adolescents (73% girls) between the ages of 12 and 17 (*M* = 15.3, *SD* = 1.35), recruited from the province of Quebec, Canada. Participants varied with respect to educational level at T1 with 1% at elementary school, 81% attending high school, 16% enrolled in college and 2% completing a professional training program or employed. Most adolescents lived in a two-parent household (65% report living with two parents; 12% with a parent and a step-parent; 18% with a single parent; and the remainder report another family arrangement as with a guardian, a foster family or in an apartment). Among the 294 participants, 71 took part in T2 and 53 had complete data on all study variables (see Table 1 for information on the *N* per variable and section 2.4.1 on missing data).

**Table 1:** Descriptive statistics (*N* = 294).

Descriptive statistics *N* = 294					
Continuous variables	*N*	Min.	Max.	*M*	SD
Age (years)	294	12.00	17.00	15.30	1.30
Hours spent engaging in physical activities in the last week (T1)	249	1.00	7.00	4.20	1.90
Self-esteem (T1)	155	3.00	30.00	19.30	5.90
Psychological Distress (T1)	213	0.00	100.0	41.10	24.40
Psychological Distress (T2)	71	0.00	95.20	43.00	25.70
Categorical variables	*N* (%)				
Gender (girls)	214 (73)				
Complete cases *N*	53				

*N* = sample; Min.: minimum; Max.: maximum; *M*: mean; SD: standard deviation.

Little test MCAR: *χ*^2^(28) = 28.611, *p* = 0.432.

### 2.3 Instruments

The number of hours spent engaging in physical activity over the past 7 days (*Hours spent engaging in physical activities;* HPA) was measured at T1 (summer 2020) using a single question from the Quebec health surveys: “In the last week (total of the last 7 days), how much time did you spend on physical activities or sports (running, cycling, scootering, ball, etc.)?”

*The Rosenberg Self-Esteem Scale* (RSES) (44, 45), administered at T1, consists of 10 items assessing a person's judgment of his or her self-worth. Participants responded on a 4-point Likert scale ranging from 1 (strongly disagree) to 4 (strongly agree), where a higher score reflects greater self-esteem. This scale is the most widely used to assess self-esteem.

*The Psychological Distress Index* (PDI) (46), administered at T1 and T2, is a 14-item questionnaire used in Quebec health surveys (47) assessing symptoms of depression, anxiety, irritability, and cognitive problems over the past two weeks. A total distress score is also provided. Participants rated each item on a 4-point Likert scale ranging from (0) “never” to (3) “very often”. Items include statements such as “Did you feel lonely”, “Did you feel any fears or worries”, “Did you feel easily upset or irritated”. A higher score reflects greater psychological distress. The IDP has excellent psychometric qualities (alphas for the current study = .87, .87, .82, .79, respectively) and has been extensively used in studies evaluating psychological distress in adolescents as well as in surveys on the health of high school students from Quebec (47).

### 2.4 Analyses

**Figure 1:**
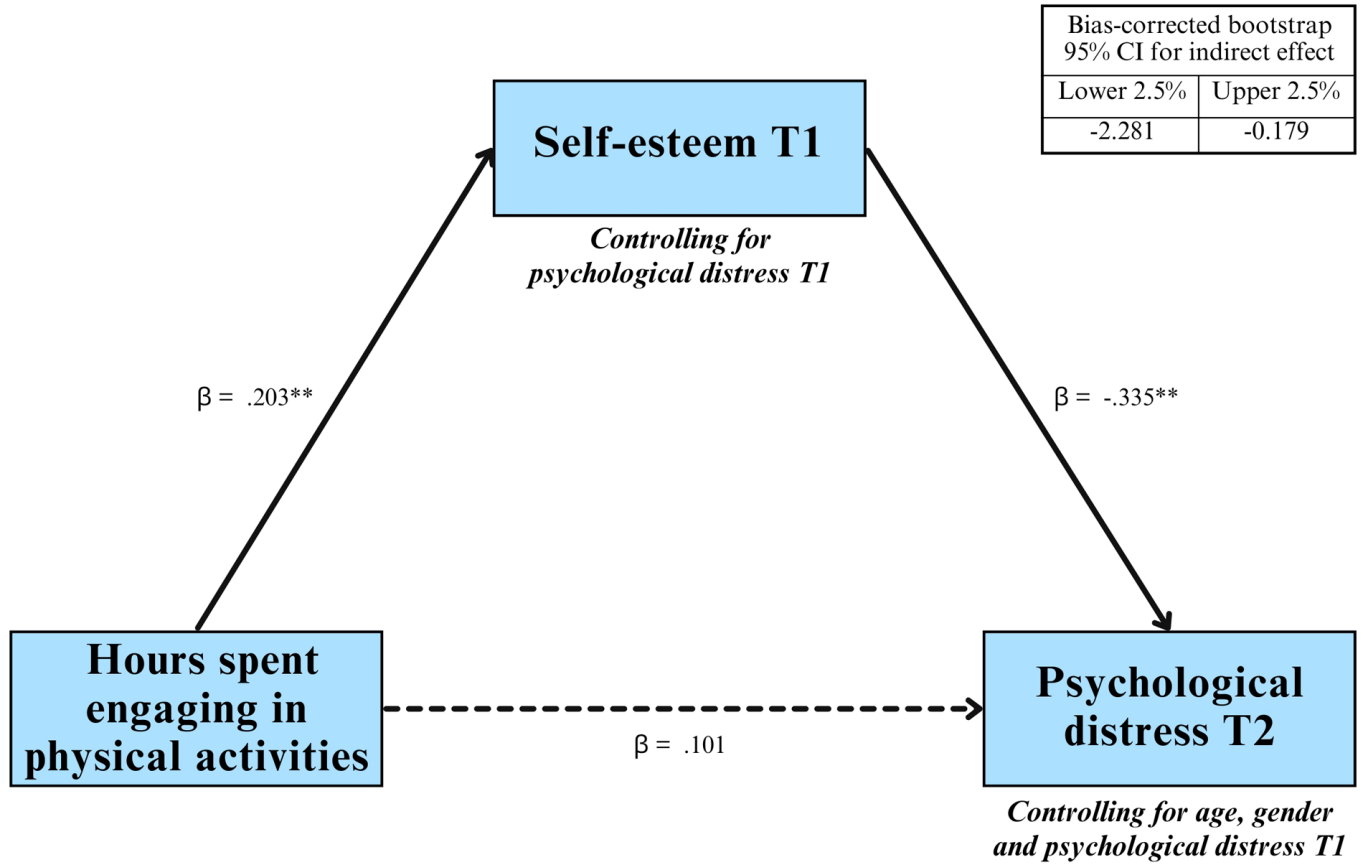
Indirect effect between hours spent engaging in physical activity and psychological distress through self-esteem. Asterisk (*) data labels indicate significant difference. **p < .01, *p < .05.

An indirect effect model was carried out using Mplus software version 8.10 (48) via a structural equation modeling (SEM)^1^. The model, presented in Figure 1, was estimated on the entire sample (*N* = 294) using the full information maximum likelihood (FIML) technique. This technique requires missing data to be at least missing at random (MAR) and enables model parameters to be estimated using the maximum information available between the variables under study. The maximum likelihood with robust standard errors (MLR) estimator was used to estimate model parameters. The indirect effect is considered significant if its bias-corrected bootstrap confidence interval does not include the value “0”. To select covariates, a *p*-value cut-off point of .25 was used (49). This decision is guided by previous work showing that a *p*-value cut-off of .05 may fail to identify important covariates (50, 51). Indeed, variables showing weak bivariate associations with outcomes may become significant predictors when considered in a more comprehensive model (49). The final model will respect the usual fit indices (52).

#### 2.4.1 Missing data

In the sample, 82% of participants had at least one missing data item on the model variables. In accordance with other longitudinal studies with a large number of missing data (53), additional statistical steps were taken in the present study to test the robustness of the model. Specifically, to assess the robustness of the FIML estimation for accounting missing data, we conducted analyses on complete cases only (*N* = 53) and using multiple imputation for missing data replacement. Imputation was carried out using the variables under study, 100 imputed data sets were estimated with software Mplus using weighted least squares with mean and variance adjusted (WLSMV) estimator. The model was then estimated on pooled imputed data sets, combining results from several imputed data sets to obtain parameter estimates and final inferences. All three strategies provide a similar pattern of results. Little's test was non-significant, which showed no evidence against data missing completely at Random (MCAR) (*χ*^2^(28) = 28.611, *p* = 0.432), making FIML an appropriate strategy. Results based on complete cases and using multiple imputation are presented in the Supplement Material (Table 3).

## 3 Results

Descriptive statistics of main study variables are presented in Table 1. On average, adolescents were physically active for 4.2 h (*SD* = 1.9) over the last 7 days, representing less than an hour a day. Bivariate correlation coefficients (Table 2) show that greater HPA, as measured by time spent engaging in physical activity over the past week, was associated with greater self-esteem. Greater self-esteem at T1 was related to less psychological distress at T2. The bivariate correlation between HPA and psychological distress at T2 was non-significant, which justifies testing an indirect link rather than mediation. Considering that psychological distress at T1, gender and age were associated to psychological distress at T2 with a *p*-value < .25, they were considered as covariables in the tested model (see Figure 1).

**Table 2:** Correlations between age, gender, hours spent engaging in physical activities, self-esteem and psychological distress.

Variables	1.	2.	3.	4.	5.
1. Age	–				
2. Gender (0 = Girl, 1 = Boy)	−.056	–			
3. Hours spent engaging in physical activities	−.203***	−.092*	–		
4. Self-Esteem (T1)	−.024	−.199**	.259***	–	
5. Psychological distress (T1)	.117*	.262***	−.147**	−.538***	–
6. Psychological distress (T2)	.180*	.203*	−.074	−.543***	.645***

Asterisk (*) data labels indicate significant difference. *p < .25, **p < .05, ***p < .01.

Results confirmed an indirect mechanism (95% C.I.: −2.281, −0.179). Namely, greater hours engaging in physical activity in the past week was related to greater self-esteem at T1 (*b* = 0.628, se = 0.229, *p* = 0.006, *β* = 0.203). Greater self-esteem at T1 was in turn related to lower psychological distress at T2 (*b* = −1.499, se = 0.475, *p* = 0.002, *β* = −0.335), controlling for distress at T1, gender, and age. Lastly, hours spent engaging in physical activity in the past week were not linked to psychological distress at T2 (*b* = 1.410, se = 1.305, *p* = 0.280, *β* = 0.101), controlling for distress at T1, gender, and age. The results thus support that physical activity is indirectly and negatively associated to psychological distress through self-esteem. We also tested the model without controlling for distress at T1 and the results provide a similar patten of results (Supplement Material Table 4).

## 4 Discussion

These results support that the practice of physical activity during a time of tumult can have mid-term benefits on mental health adjustment through its association with self-esteem. Indeed, the practice of physical activity in the past 7 days was linked to greater self-esteem among the participants of the study. Participants with greater self-esteem then presented fewer symptoms of psychological distress 6 months later, during the prolonged period of uncertainty of the COVID-19 pandemic.

Using a longitudinal design, the present findings significantly add to the breath of empirical evidence supporting the benefits of physical activity in promoting greater psychological adjustment over time though greater self-esteem. These findings echo the assumption that physical activity and participation in sports are minimally invasive “interventions” (54) with significant benefits for mental health. As such, ensuring that youth maintain opportunities to participate in sports and physical activities by providing continued access to sports facilities and adapting, if necessary, organized sports activities during times of crisis and turmoil seems essential as it is linked to their self-esteem, a factor associated with better psychological health over time (22, 31, 32, 42). Physical activity can lead to a sense of personal competence and gratification through physical achievement, which in turn can boost self-esteem. In turn, improved self-esteem can ensures better mental health; a feeling of being a person who has something worthwhile to offer could protect against mental health difficulties.

A major contribution of the present study is the assessment of the indirect model while controlling for initial distress among adolescents. This allowed us to tease apart the unique contribution of physical activity and self-esteem in predicting psychological distress over time. Nonetheless, certain limitations need to be addressed. First, nearly two-thirds of participants were girls, limiting the generalizability of study findings. This over-representation of adolescent girls is typical within social-sciences research and has also been the case in studies using social medias as a recruitment strategy (55). Nevertheless, we controlled for gender in the present study, mitigating the impact of this limitation. Additional research with a larger sample and greater gender representation is warranted to replicate the present findings. Participant attrition was also high in the present study. However, the two additional analyses carried out (results based on complete cases and using multiple imputation) add to the validity of the identified indirect model. Moreover, given that physical activity and self-esteem were assessed at the same time point, we cannot speak to the causal nature of their association. Indeed, while the practice of physical activity is linked to better self-esteem, the opposite may also true: young people with higher self-esteem could be more likely to engage in physical activity. We also know that adolescents with good mental health are more likely to engage in physical activity, supporting the idea of a bidirectional relationship between physical activity and mental health (56). The present findings must therefore be interpreted with caution and within the scope of the study's design. Nevertheless, the findings highlight how physical activity and self-esteem are related to psychological distress, with physical activity influencing distress indirectly through self-esteem.

To conclude, while this study was conducted during the COVID-19 pandemic, findings are relevant beyond this scope as lessons learned can be reinvested in a host of other potential situations of adversity faced by young people (e.g., school violence, family displacement due to natural disasters, etc.) to ensure their psychological well-being. For instance, schools and community centers should encourage youth engagement in physical activity as it is beneficial for physical and psychological health, in both the short and longer term. Decision makers should also invest in providing youth with green spaces to promote outdoor activities, should future social distancing measures be implemented.

## Data Availability

The datasets presented in this article are not readily available because in compliance with the requirements related to the consent obtained and approved by the research ethics committee and the sensitive nature of the information provided by the participants, the data are not available. Requests to access the datasets should be directed to catherine.laurier@usherbrooke.ca.

## Footnotes

^1^As the relation between HPA and psychological distress at T2 was not significant, the mediation hypothesis was not feasible. Although closely related, an indirect model is not the same as a mediation model. An indirect model is a statistical model that shows that an independent variable influences a dependent variable in a non-direct way, i.e. via one or more intermediate variables. While mediation is a more specific concept, referring to a causal process in which a significant relationship between two variables (X and Y) is explained by a third variable in between, every mediation model is an indirect model, but the reverse is not always true, as in the present study.
